# MotorPlex provides accurate variant detection across large muscle genes both in single myopathic patients and in pools of DNA samples

**DOI:** 10.1186/s40478-014-0100-3

**Published:** 2014-09-11

**Authors:** Marco Savarese, Giuseppina Di Fruscio, Margherita Mutarelli, Annalaura Torella, Francesca Magri, Filippo Maria Santorelli, Giacomo Pietro Comi, Claudio Bruno, Vincenzo Nigro

**Affiliations:** 1 Laboratorio di Genetica Medica, Dipartimento di Biochimica, Biofisica e Patologia generale, Seconda Università degli Studi di Napoli, Napoli, Italy; 2 Telethon Institute of Genetics and Medicine, Napoli, Italy; 3 Dino Ferrari Center, IRCCS Ca’ Granda Ospedale Maggiore Policlinico, Neuroscience Section, Dipartimento di Fisiopatologia medico-chirurgica e dei trapianti, Università di Milano, Milano, Italy; 4 Molecular Medicine and Neuromuscular Lab, IRCCS Stella Maris, Pisa, Italy; 5 Centro di Miologia e Patologie Neurodegenerative, IRCCS Istituto Giannina Gaslini, Genova, Italy

**Keywords:** Next generation sequencing, Myopathies, Target sequencing, Pooling, Muscular dystrophies

## Abstract

Mutations in ~100 genes cause muscle diseases with complex and often unexplained genotype/phenotype correlations. Next-generation sequencing studies identify a greater-than-expected number of genetic variations in the human genome. This suggests that existing clinical monogenic testing systematically miss very relevant information.

We have created a core panel of genes that cause all known forms of nonsyndromic muscle disorders (MotorPlex). It comprises 93 *loci*, among which are the largest and most complex human genes, such as *TTN*, *RYR1*, *NEB* and *DMD*. MotorPlex captures at least 99.2% of 2,544 exons with a very accurate and uniform coverage. This quality is highlighted by the discovery of 20-30% more variations in comparison with whole exome sequencing. The coverage homogeneity has also made feasible to apply a cost-effective pooled sequencing strategy while maintaining optimal sensitivity and specificity.

We studied 177 unresolved cases of myopathies for which the best candidate genes were previously excluded. We have identified known pathogenic variants in 52 patients and potential causative ones in further 56 patients. We have also discovered 23 patients showing multiple true disease-associated variants suggesting complex inheritance. Moreover, we frequently detected other nonsynonymous variants of unknown significance in the largest muscle genes. Cost-effective combinatorial pools of DNA samples were similarly accurate (97-99%).

MotorPlex is a very robust platform that overcomes for power, costs, speed, sensitivity and specificity the gene-by-gene strategy. The applicability of pooling makes this tool affordable for the screening of genetic variability of muscle genes also in a larger population. We consider that our strategy can have much broader applications.

**Electronic supplementary material:**

The online version of this article (doi:10.1186/s40478-014-0100-3) contains supplementary material, which is available to authorized users.

## Introduction

Muscle genetic disorders comprise about 100 different genetic conditions [1],[2], characterized by a clinical, genetic and biochemical heterogeneity. The molecular diagnosis for myopathic patients is crucial for genetic counseling, for prognosis and for available and forthcoming mutation-specific treatments [3]-[5]. In addition, patients that share the same mutation may have a different type of muscle affection with the selective involvement of other muscle compartments or myocardial damage. Thus, the primary defect may be modified or not by additional and variable elements that may be genetic or not. The most severe cases of congenital or childhood-onset myopathies often result from mutations in genes encoding proteins belonging to common pathways [6]. To provide a clue to address genetic testing, a muscle biopsy is often required that may be useful, but not well accepted by patients. The single gene testing can be diagnostic only in patients with most recognizable disorders. In unspecific cases of muscular diseases, however, no effective methodology has been developed for the parallel testing of all disease genes identified so far [7].

Next-generation sequencing (NGS) is changing our view of biology and medicine allowing the large-scale calling of small variations in DNA sequences [8]. In the last few years, the whole-exome sequencing (WES) and whole-genome sequencing (WGS) have received widespread recognition as universal tests for the discovery of novel causes of Mendelian disorders in families [9]. The power to discover a novel Mendelian condition increases with the family size, even if successful studies, identifying novel disease genes from multiple small families with the same phenotype, have been published [10]. Structural and copy number variations are not well detected by NGS technologies [11]-[14]. However, the WES/WGS use for the clinical testing of isolated cases is still debated. First, there are ethical issues linked to the management of the incidental findings [15]. The second limitation is given by the practical problem that the coverage is usually too low for clinical diagnosis. Hence the cost-effectiveness is reduced, considering that WES/WGS may require either numerous validation procedures, mainly based on conventional PCR and Sanger sequencing reactions [16]. Innovative strategies of clinical exome sequencing at high coverage have been described [17], but the cost for a single patient is still too high for routine diagnosis. Thus, there is still space for targeted strategies [18] and the HaloPlex Target Enrichment System [19] represents an innovative technology for targeting, since it uses a combination of eight different enzyme restriction followed by probe capture. It permits a single-tube target amplification and one can accurately predict the precise sequence coverage in advance. We have developed a NGS targeting workflow as a single testing methodology for the diagnosis of genetic myopathies that we named Motorplex. Here we demonstrate the high sensitivity and specificity of Motorplex. We challenged our platform against complex DNA pools. Even with this complexity, Motorplex kept producing reliable data with high sensitivity and specificity values. Furthermore, pooling reduced the cost of the entire analysis at negligible values, implementing applications for large studies of populations [16],[20].

## Materials and methods

### Patients

Encrypted DNA samples from patients with clinical diagnosis of nonspecific myopathies, congenital myopathy, proximal muscle weakness or limb-girdle muscular dystrophy (LGMD) were included. The Italian Networks of Congenital Myopathies (coordinated by C.B. and F.M.S.) of LGMD (by F.M. and G.P.C.) were involved together with a large number of other single clinical centers. We asked all them the possibility to share more clinical and laboratory findings, when necessary. We also requested to provide information on familial segregation and previous negative genetic tests. Internal patients signed a written informed consent, according to the guidelines of Telethon Italy and approved by the Ethics Committee of the “Seconda Università degli Studi di Napoli”, Naples, Italy.

DNA samples were extracted using standard procedures. DNA quality and quantity were assessed using both spectrophotometric (Nanodrop ND 1000, Thermo Scientific Inc., Rockford, IL, USA) and fluorometry-based (Qubit 2.0 Fluorometer, Life Technologies, Carlsbad, CA, USA) methods.

### In silico design of MotorPlex

We included in the design all the 93 genes that are universally considered as genetic causes of nonsyndromic myopathies (Additional file 1: Table S1). In particular, we only selected genes determining a primary skeletal muscle disease, such as underlying muscular dystrophies, congenital myopathies, metabolic myopathies, congenital muscular dystrophies, Emery-Dreifuss muscular dystrophy, etc. We therefore excluded loci associated with other neuromuscular and neurological disorders such as congenital myasthenias, myotonic dystrophy, spinal muscular atrophy, ataxias, neuropathies, or paraplegias for which differential diagnosis may be clinically possible. For each locus, all predicted exons and at least ten flanking nucleotides were always included in the electronic design by the custom NGS Agilent SureDesign webtool. Setting the sequence length at 100×2 nucleotides, the predicted target size amounted to 2,544 regions and 493.598kb. Around 20% of the target is represented by TTN coding regions.

### NGS workflow

For library preparation of single samples, we followed the manufacturer’s instructions (HaloPlex Target Enrichment System For Illumina Sequencing, Protocol version D, August 2012, Agilent Technologies, Santa Clara, CA, USA). We started using 200ng of genomic DNA and strictly followed the protocol, with the exception that restricted fragments were hybridized for at least 16–24 hours to the specific probes. After the capture of biotinylated target DNA, using streptavidin beads, nicks in the circularized fragments were closed by a ligase. Finally, the captured target DNA was eluted by NaOH and amplified by PCR. Amplified target molecules were purified using Agencourt AMPure XP beads (Beckman Coulter Genomics, Bernried am Starnberger See, Germany).

The enriched target DNA in each library sample was validated and quantified by microfluidics analysis using the Bioanalyzer High Sensitivity DNA Assay kit (Agilent Technologies) and the 2100 Bioanalyzer with the 2100 Expert Software. Usually 20 individual samples were run in a single lane (250M reads), generating 100-bp paired end reads.

For Pool-Seq experiments, equimolar pools of 5 or 16 DNA samples (*detector* and *scouting* pools) were created and 200ng of each pool was used for the HaloPlex enrichment strategy. Sixteen detector and five scouting pools were usually run in a single HiSeq1000 lane.

### Targeted sequencing analysis

The libraries were sequenced using the HiSeq1000 system (Illumina inc., San Diego, CA, USA). The generated sequences were analyzed using an in-house pipeline designed to automate the analysis workflow, composed by modules performing every step using the appropriate tools available to the scientific community or developed in-house [21]. Paired sequencing reads were aligned to the reference genome (UCSC, hg19 build) using BWA [22], sorted with Picard (http://picard.sourceforge.net) and locally realigned around insertions-deletions with Genome Analysis Toolkit (GATK) [23]. The UnifiedGenotyper algorithm of GATK was used for SNV and small insertions-deletions (ins-del) calling, with parameters adapted to the Haloplex-generated sequences. The analysis of pools was performed with UnifiedGenotyper as well, adapting the ploidy parameter to the number of chromosomes present in the samples (10 for the *detector* and 32 for the *scout* pools) and the minimal ins-del fraction parameter accordingly. The called SNV and ins-del variants produced with both platforms were annotated using ANNOVAR [24] with: the relative position in genes using RefSeq [25] gene model, amino acid change, presence in dbSNP v137 [26], frequency in NHLBI Exome Variant Server (http://evs.gs.washington.edu/EVS) and 1000 genomes large scale projects [27], multiple cross-species conservation [28],[29] and prediction scores of damaging on protein activity [30]-[33]. The annotated variants were then imported into the internal variation database, which stores all the variations found in the re-sequencing projects performed so far in our institute. The database was then queried to generate the filtered list of variations and the internal database frequency in samples with unrelated phenotype was used as further annotation and filtering criteria. The alignments at candidate positions were visually inspected using the Integrative genomics viewer (IGV) [34]. We selected from the database the non-synonymous SNVs and ins-del, with a frequency lower than 2%, which was followed by manual inspection and further filtering criteria based on the presence in unrelated samples of the database, on the presence in the other samples of the Motorplex experiment and on the conservation of the mutations, with a final selection of rare, possibly causative, variations per individual.

## Results

### Validation study of MotorPlex

**Figure 1:**
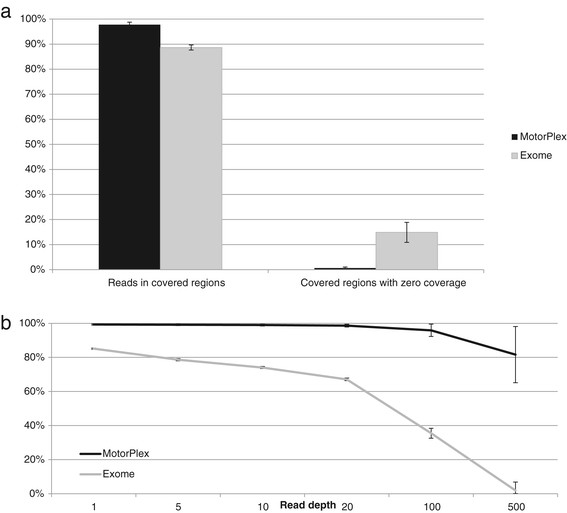
**A comparison between MotorPlex and a Whole Exome strategy** (**WES**) **demonstrates the better performance of the targeted strategy. (a)** 97.75% of reads generated in a MotorPlex experiment fall in the regions of interest and only 0.67% of targeted regions are not sequenced. On the contrary, for WES 88.66% of reads are on target and 14.89% of targeted exons are not effectively covered. **(b)** The percentage of targeted regions covered at high depth by MotorPlex is higher than that obtained by WES. In particular, 96.01% and 81.6% of regions are, respectively, covered at 100x and 200x by using MotorPlex versus 35.49% and 1.90% by WES.

To design MotorPlex we used a straightforward procedure. Briefly, disease genes causing a muscular phenotype, including the biggest genes of the human genome, like titin (*TTN*) or dystrophin (*DMD*), were selected. The target sequences, corresponding to 0.5Mbp were enriched by the HaloPlex system (see Materials and methods). To validate MotorPlex, we created a training set of twenty DNA samples belonging to patients (15 males and 5 females) affected by different forms of limb-girdle muscular dystrophy or congenital myopathy (Additional file 2: Table S2) and compared with data from whole exome sequencing (WES) (Figure 1). For each sample, about 98% of reads generated (Figure 1a and Additional file 3: Table S4) were on target (compared to 88% obtained by WES) and fewer than 0.5% of targeted regions were not covered (about 15% of human exons are not analyzed by WES, Additional file 4: Figure S1). Moreover, more than 95% of targeted nucleotides were read at a 100× depth and a 500× depth was obtained for 80% of these; on the contrary, by performing a WES analysis, fewer than 70% of exons were covered at 20× (Figure 1b). From previous amplicon Sanger sequencing from these samples, we knew about 84 variants in 17 different genes (Additional file 5: Table S3). All these known variants were correctly called and no additional change was seen within the sequenced target (100% sensitivity and specificity). Moreover, to assess the reproducibility of the targeted enrichment and the subsequent NGS workflow, the same sample (43U) was analyzed twice. After filtering, variants were always confirmed, including the putative causative one (Table 1). Outside the Sanger coverage, 4,991 additional variations were called (Additional file 6: Table S5).

**Table 1:** **List of pathogenic variants**

Sample ID	Sex	Clinical diagnosis	Inheritance	Histopathologic features	Variant(s)					
Single1	M	CM	Sp	c.n.	DNM2	chr19:10934538*	c.1856 C>G	p.S619W	het	c.n.^sr1^
Single3	M	LGMD	Sp	m.f.	CAPN3	chr15:42695076*	c.1621 C>T	p.R541W	het	LGMD^sr2^
					CAPN3	chr15:42682142*	c.802-9G>A	spl.	het	LGMD^sr3^
Single6	M	LGMD	Rec	m.f.	FKRP	chr19:47259458	c.751G>T	p.A251S	het	
					FKRP	chr19:47259758	c.G1051C	p.A351P	het	
Single8	M	LGMD	Sp	n.a.	DYSF	chr2:71838708	c.4119 C>A	p.N1373K	het	
					DYSF	chr2:71762413	c.1369G>A	p.E457K	het	
Single15	F	LGMD/CM	Sp	d.f.	SYNE2	chr14:64688329	c.663G>A	p.W221X	het	
Single16	M	LGMD/DCM	Sp	d.f.	SGCG	chr13:23869573*	c.525 delT	p.F175L fsX20	hom	LGMD^sr4^
					LDB3	chr10:88446830*	c.349G>A	p.D117N	het	DCM^sr5^
Single19	M	LGMD	Sp	m.f.	RYR1	chr19:39062797*	c.13885G>A	p.V4629M	het	CM^sr6^
Single20	M	LGMD/DCM	Rec	c.n.	RYR1	chr19:39009932*	c.10097G>A	p.R3366H	het	Multiminicore^sr7^
					RYR1	chr19:38973933*	c.4711 A>G	p.I1571V	het	MH^sr8^
					RYR1	chr19:39034191*	c.11798A>G	p.Y3933C	het	MH^sr9^
					RYR1	chr19:38942453	c.G1172C	p.R391P	het	
					DES	chr2:220284876*	c.638 C>T	p.A213V	het	DCM^10^
1/17s	F	CM	Sp	c.n.	TTN	chr2:179452695*	c.63439G>A	p.A21157T	het	ARVD^sr11^
					TTN	chr2:179496025	c.G43750T	p.G14584X	het	
					TTN	chr2:179392277*	c.107576T>C	p.M35859T	het	ARVD^sr11^
1/21s	M	LGMD	n.a.	n.a.	SGCA	chr17:48246607*	c.739G>A	p.247V>M	het	LGMD^sr12^
					SGCA	chr17:48245758*	c.409G>A	p.E137K	het	LGMD^sr13^
2/17s	F	CM	Sp	cftdm	MYH7	chr14:23886406	c.T4475C	p.L1492P	het	
2/20s	M	LGMD	n.a.	n.a.	POMT2	chr14:77745129*	c.1975 C>T	p.659 R>W	het	CMD^sr14^
					POMT2	chr14:77769283*	c.551 C>T	p.T184M	het	LGMD^sr15^
3/20s	F	LGMD	Sp	cftdm	TPM2	chr9:35689792*	c.20_22delAGA	p.7Kdel	het	CM^sr16^
4/17s	M	LGMD	Rec	c.n.	ANO5	chr11:22242646*	ANO5:c.191dupA	p.64N>Kfs*15	hom	LGMD^sr17^
4/18s	M	LGMD	Sp	vacuoles	DNAJB6	chr7:157175006	c.413G>A	p.G138E	het	
5/17s	M	LGMD/DCM	Sp	m.f.	MYOT	chr5:137213267	c.591delTG	p.199F>S fsX3	het	
5/21s	M	LGMD	Sp	c.n.	CAV3	chr3:8787288*	c.191C>G	p.T64S	het	HCM^sr18^
6/20s	M	LGMD	Sp	d.f.	ACADVL	chr17:7127330*	c.G1376A	p.R459Q	het	VLCAD^sr19^
					ACADVL	chr17:7128130	c.C754T	p.A585V	het	
7/17s	M	LGMD	Sp	m.f.	CAPN3	chr15:42702843*	c.2242 C>T	p.R748X	het	LGMD^sr20^
					CAPN3	chr15:42693952*	c.1468 C>T	p.R490W	het	LGMD^sr21^
7/20s	F	LGMD	Sp	d.f.	LMNA	chr1:156100408*	c.357 C>T	p.R119R (spl.)	het	EDMD^sr22^
8/19s	M	LGMD	n.a.	d.f.	DNAJB6	chr7:157155959	c.C170T	p.S57L	het	
10/17s	F	CM	Sp	m.f.	MYH7	chr14:23886518	c.G4363T	p.E1455X	het	
10/21s	M	LGMD/FSHD	Dom	d.f.	SMCHD1	chr18:2700849*	c.C1580T	p.T527M	het	FSHD^sr23^
11/18s	M	CM	Sp	nemaline	NEB	chr2:152447860	c.6915+2T>C	spl.	het	
					NEB	chr2:152553662	c.C1470T	p.D490D (spl.?)	het	
12/18s	F	CM	Sp	cftdm	MYH7	chr14:23882063	c.G5808C	p.X1936Y	het	
12/21s	F	LGMD	Sp	d.f.	PYGM	chr11:64519958	c.A1537G	p.I513V	het	
					PYGM	chr11:64514809*	c.C2199G	p.Y733X	het	McArdle^sr24^
13/20s	M	LGMD	Rec	n.a.	LAMA2	chr6:129722399*	c.C5476T	p.R1826X	het	LGMD^sr25^
					LAMA2	chr6:129571264	c.1791_1793del AGT	p.598 del V	het	
13/21s	M	LGMD	Sp	d.f.	SGCG	chr13:23898652*	c.848G>A	p.C283Y	hom	LGMD^sr26^
14/20s	F	LGMD	n.a.	n.a.	CAPN3	chr15:42686485*	c.1061T>G	p.V354G	het	LGMD^sr21^
					CAPN3	chr15:42689077	c.1193+2T>C	spl.	het	
14/18s	M	LGMD	n.a.	d.f.	DMD	chrX:32360366*	c.G5773T	p.E1925X	hem	Duchenne^sr27^
15/19s	M	CM	Sp	multiminicores	MYH7	chr14:23885313*	c.4850_4852del	p.1617 del K	het	Distal^sr28^
16/18s	M	LGMD	Sp	no alterations	CAPN3	chr15:42691746*	c.1250 C>T	p.T417M	hom	LGMD^sr29^
16/20s	M	CM	Sp	cftdm	TTN	chr2:179431175	c.C79684T	p.R26562X	het	
					TTN	chr2:179526510	c.A39019T	p.K13007X	het	
16/21s	F	CM	Dom	n.a.	TPM2	chr9:35685541*	c.A382G	p.K128E	het	CFTD^sr30^
23/38s	M	CM	Sp	cftdm	RYR1	chr19:38959672	c.3449delG	p.C1150fs	het	
					RYR1	chr19:38985186	c.6469G>A	p.E2157K	het	
					RYR1	chr19:39003108*	c.9457G>A	p.G3153R	het	MH^sr31^
23/41s	M	CM	Sp	m.f.	RYR1	chr19:38990637*	c.G7304T	p.R2435L	hom	CCD^sr32^
24/42s	F	CM	n.a.	n.a.	ACTA1	chr1:229567867*	c.G682C	p.E228Q	het	Nemaline^sr33^
25/38s	M	CM	Sp	cftdm	CRYAB	chr11:111779520	c.A496T	p.K166X	het	
25/39s	F	CM	Dom	c.n.	RYR1	chr19:39075614*	c.14678G>A	p.R4893Q	het	CCD^sr34^
25/41s	F	CM	n.a.	n.a.	MYH7	chr14:23886750	c.G4315C	p.A1439P	het	
28/39s	F	CM	Dom	minicore	MYH7	chr14:23885313*	c.4850_4852del	p.1617del K	het	Distal^sr28^
28/41s	M	CM	Sp	c.n.	MTM1	chrX:149831996*	c.C1558T	p.R520X	hem	Myotubular^sr35^
29/41s	F	CM	Rec	n.a.	NEB	chr2:152387617	c.21628-2A>T	spl.	het	
					NEB	chr2:152541300	c.C2827T	p.Q943X	het	
30/42s	F	CM	Rec	cftdm	RYR1	chr19:38948185*	c.C1840T	p.R614C	het	MH^sr36^
					RYR1	chr19:38959747	c.G3523A	p.E1175K	het	
31/42s	F	CM	Rec	nemaline	NEB	chr2:152471093	c.11298_11300delTAC	p.Y3766del	hom	
32/41s	M	CM	Dom	c.n.	MTM1	chrX:149826390	c.1150 C>T	p.Q384X	het	
32/42s	F	CM	Dom	minicore	DNM2	chr19:10939917	c.C2252A	p.T751N	het	
33/41s	M	CM	Rec	nemaline	NEB	chr2:152370944	c.23122-2A>G	spl.	het	
					NEB	chr2:152544037	c.A2533G	p.K845E	het	
36/42s	M	CM	Dom	n.a.	RYR1	chr19:39075629*	c.T14693C	p.I4898T	het	CCD^sr37^
37/39s	M	LGMD	Sp	d.f.	DMD	chrX:32841417*	c.T328C	p.W110R	hem	Becker^sr38^
37/40s	F	LGMD	Sp	n.a.	SYNE2	chr14:64676751*	c.C18632T	p.T6211M	het	EDMD^sr39^
37/41s	F	CM	Dom	m.f.	MTM1	chrX:149826390	c.1150 C>T	p.Q384X	het	

*Already reported. For references, see Additional file 10.

### Validation study of double-check pooling

**Figure 2:**
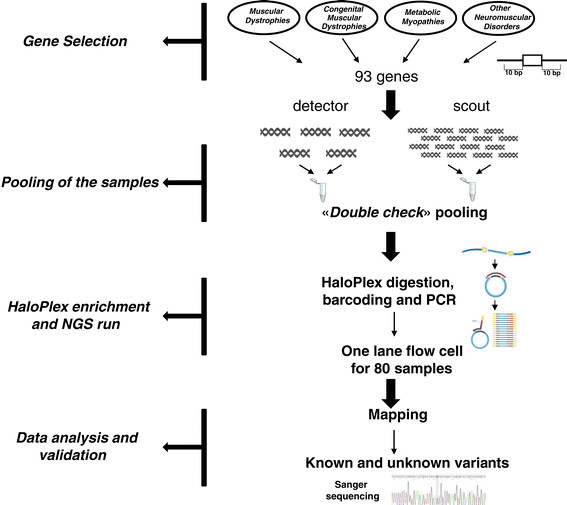
**NGS targeting workflow.** Ninety-three disease genes causing a muscular phenotype were selected. To cover all their exons and the ten flanking bases, an enrichment strategy, based on HaloPlex system, was designed. DNA samples of 80 patients were analyzed twice in an independent manner, using a combinatorial pooling scheme. As requested by HaloPlex protocol, DNA samples were digested, barcoded and amplified. The 80 samples were run at the same time in a single lane of the flow cell of HiSeq 1000. The following data analysis allowed us to detect putative causative variants validated by Sanger sequencing.

To challenge MotorPlex to be applied to large studies on thousands of patients and/or to detect mosaic mutations, we designed a combinatorial pooling strategy. After some initial attempts with pools of identical sizes, we changed our strategy. The general arrangement was to have the same sample in two different independent pools, composed of two exclusive combinations of samples (Figure 2). This permitted us to identify both the rare variations and the sample mutated. In particular, the pools were organized in two groups: the “detector pool” only containing five samples (10 alleles) that had the purpose of detecting variations with the optimal sensitivity and the “scout pool” composed of 16 samples (32 alleles) that confirmed the variation(s) and attribute them univocally to distinct DNA samples (Additional file 7: Figure S2; Additional file 8, Table S6). We paid attention each time to include the index cases alone, excluding related family members.

To validate this arrangement, we selected five samples that we previously sequenced individually and called 1,235 variations. We pooled them in the same detector pool (P9) and then reanalyzed in different scout pools. Impressively, in pool P9 we called 1,232/1,235 variations belonging to the individual samples, calculating the sensitivity value at 99.8%. The three missing variations (an insertion in RRM2B and two point variants in TTN) were located in regions with lower coverage. On the contrary, no variation was called in pool 9 in addition to those of individual samples, demonstrating the absence of false positives and artefacts due to the pooling strategy. Another two samples from the training set were inserted in another two detector pools, showing similar results.

We then confirmed 223/230 (97%) variations tested by Sanger sequencing, thus providing the specificity value of the method. Moreover, the combined use of detector pools and scout pools allowed us to “clean” the results. 50% of off target variations (n=1,291), in fact, were not called in the scout pools and were easily filtered off during bionformatic analysis. In addition, about 25% of variants in low covered regions (<500 total reads), representing in a large percentage false positive calls, were similarly filtered off because they were not detected in the scout pools (Additional file 9: Figure S3).

### Variants and interpretation

The targeted analysis of 93 genes showed a total of 23,109 rare variants (<0.01 frequency) in 173 patients (1.4 variants/gene/patient). To provide a preliminary interpretation in relationship with the clinical suspicion, we set bioinformatic filters that weigh the variant class (missense, indel, stopgain or stoploss), the calculated frequency in public and internal databases and the annotation as causative variants. Finally, we reconsidered critically the correspondence with the clinical presentation, the age at onset and the segregation in familial cases.

In detail, we identified 52 patients (52/177=29%) with variants of likely pathogenicity or predicted to affect function (Table 1 and Additional file 10): most of them (38/52=73%) had known or truncating variants (indel, stopgtain or stoploss). Five patients (5/52=9.6%) showed a novel variant in addition to a pathogenic allele in a recessive gene. The remaining samples (9/52=17%) had novel variants that are predicted to affect function in genes fitting with the clinical suspicion.

In other 56 samples (56/177=32%), we identified potential causative variants (Table 2 and Additional file 10). In these cases, there was only a partial correspondence with the clinical phenotype. For example, a number of variants had been previously associated with cardiomyopathy, but their pathogenic role in congenital myopathy or in LGMDs was not yet established. To the group belong patients having two rare variants in *TTN* gene or at least one variant in *COL6A1*, *COL6A2*, *COL6A3*, *SYNE1*, *SYNE2* and *FLNC* genes. These molecular findings in these 56 samples were not considered strictly disease-causing and further tests are required.

**Table 2:** **Variants of unknown significance** (**Vous**)

Sample ID	Sex	Clinical diagnosis	Inheritance	Histopathologic features	Variant(s)					
Single7	M	LGMD/EDMD	Rec	d.f.	NEB	chr2:152468776	c.A11729G	p.D3910G	het	
					NEB	chr2:152495898	c.C8890T spl.	p.R2964C	het	
					COL6A2	chr21:47552071	c.2665 C>T	p.Q889X	het	
Single9	M	LGMD	n.a.	m.f.	RYR1	chr19:38986923*	c.6617 C>T	p.T2206M	het	MH^sr40^
Single13	M	CM	Sp	n.a.	LAMA2	chr6:129687396*	c.G4750G>A	p.G1584S	het	LGMD^sr41^
					LAMA2	chr6:129775423	c.6697G>A	p.V2233I	het	
					NEB	chr2:152506812	c.C7309T	p.R2437W	het	
					NEB	chr2:152512781	c.T6381A	p.D2127E	het	
Single14	F	LGMD	Sp	d.f.	COL6A3	chr2:238249316	c.C8243T	p.P2748L	het	
					COL6A3	chr2:238289767	c.A1688G	p.D563G	het	
Single18	M	CM	n.a.	n.a.	HSPG2	chr1:22176684	c.7296 A>T	spl.	het	
					HSPG2	chr1:22200473	c.3688G>A	p.G1230S	het	
1/18s	M	CM	Sp	c.n.	RYR1	chr19:38990340	c.G7093A	p.G2365R	het	
					RYR1	chr19:39018347*	c.G10747C	p.E3583Q	het	MH^sr42^
2/19s	M	LGMD/DCM	Sp	d.f.	NEB	chr2:152404851	c.G20128A	p.V6710I	het	
					NEB	chr2:152534216	c.C3637T	p.T1213M	het	
3/17s	F	LGMD	Sp	cftdm	SYNE2	chr14:64407373	c.A121G	p.I41V	het	
4/21s	M	LGMD	Sp	d.f.	MYH7	chr14:23882979*	c.A5779T	p.I1927F	het	HCM^sr43^
					FLNC	chr7:128487762	c.C4300T	p.R1434C	het	
5/18s	M	LGMD	n.a.	n.a.	TTN	chr2:179393000	c.107377+1G>A	spl.	het	
					TTN	chr2:179441932	c.C69130T	p.P23044S	het	
5/19s	F	CM	n.a.	n.a.	TTN	chr2:179439491	c.C71368T	p.R23790C	het	
					TTN	chr2:179596569	c.G17033A	p.R5678Q	het	
5/20s	M	LGMD	Sp	d.f.	COL6a3	chr2:238283289*	c.C3445T	p.R1149W	het	AVSD^sr44^
					COL6a3	chr2:238296516	c.C1021T	p.R341C	het	
					NEB	chr2:152476125	c.G10712C	p.R3571P	het	
					NEB	chr2:152580847	c.A539G	p.K180R	het	
6/21s	M	CM	Dom	cftdm	SYNE1	chr6:152776709	c.C2744T	p.T915I	het	
					SYNE2	chr14:64468677	c.C3664T	p.R1222W	het	
7/19s	M	CM	Sp	cftdm	COL6A3	chr2:238287746*	c.G2030A	p.R677H	het	Bethlem^sr45^
7/21s	M	LGMD	Sp	normal	TTN	chr2:179500777	c.G41521A	p.D13841N	het	
					TTN	chr2:179615278	c.T11849C	p.I3950T	het	
8/20s	F	LGMD	Sp	d.f.	COL6A3	chr2:238253701	c.C7162T	p.P2388S (spl.)	het	
8/21s	M	LGMD	Sp	d.f.	SMCHD1	chr18:2740713	c.C3527T	p.T1176I	het	
10/18s	F	LGMD	n.a.	n.a.	RYR	chr19:39034191*	c.A11798G	p.Y3933C	het	MH^sr9^
10/19s	F	LGMD	Sp	d.f.	RYR	chr19:38990359*	c.A7112G	p.E2371G	het	MH^sr31^
10/21s	M	LGMD	Sp	d.f.	SMCHD1	chr18:2700849	c.C1580T	p.T527M	het	
11/17s	M	LGMD	Sp	T1FP	FHL1	chrX:135278980	c.T19C	p.S7P	het	
11/19s	M	LGMD	Dom	m.f.	MYH2	chr17:10446451	c.A769G	p.T257A	het	
11/20s	M	LGMD	Sp	normal	FLNC	chr7:128482964	c.C2506T	p.P836S	het	
12/19s	M	LGMD	Sp	d.f.	COL6A2	chr21:47545454	c.T1892C	p.F631S	het	
13/18s	M	CM	Sp	cftdm and multiminicore	MYBPC2	chr11:47356715*	c.C2783T	p.S928L	het	HCM^sr46^
					SYNE2	chr14:64447727	c.A1672C	p.K558Q	het	
14/21s	M	LGMD	Sp	d.f.	RYR1	chr19:39076763	c.C14901G	p.D4967E	het	
					RYR1	chr19:39076777	c.C14915T	p.T4972I	het	
15/20s	M	LGMD	Sp	normal	LDB3	chr10:88492723	c.T2174A	p.I725N	het	
15/21s	F	CM	Sp	central core	PHKA1	chrX:71840734	c.G1978A	p.V660I	het	
					SYNE1	chr6:152746618	c.C5165T	p.S1722L	het	
					SYNE2	chr14:64548224	c.A11410G	p.T3804A	het	
23/40s	M	CM	n.a.	c.n.	TMEM43	chr3:14175304	c.C578T	p.S193L	het	
					MYBPC3	chr11:47364189*	c.G1564A	p.A522T	het	HCM^sr47^
24/38s	M	CM	Sp	cftdm	TTN	chr2:179559591	c.G31313A	p.R10438Q	het	
					TTN	chr2:179586762	c.C22628T	p.P7543L	het	
					FLNC	chr7:128475627	c.C600T	p.P200P spl.	het	
24/39s	M	CM	n.a.	n.a.	FLNC	chr7:128492888	c.C6011T	p.S2004F	het	
24/41s	F	CM	n.a.	n.a.	TTN	chr2:179495045	c.A44204G	p.N14735S	het	
					TTN	chr2:179586756	c.G22634A	p.R7545Q	het	
25/40s	M	CM	Sp	nemaline	FLNC	chr7:128494538	c.G6799A	p.V2267I	het	
25/42s	M	CM	n.a.	cftdm	RYR1	chr19:38986890	c.C6584T	p.P2195L	het	
26/39s	M	CM	Sp	core miopathy	TTN	chr2:179431924	c.T78935C	p.L26312P	het	
					TTN	chr2:179614124	c.A13003G	p.R4335G	het	
26/41s	M	CM	n.a.	n.a.	DYSF	chr2:71740851*	c.G463A	p.G155R	het	LGMD^sr48^
					DYSF	chr2:71827853	c.C3724T	p.R1242C	het	
26/42s	M	CM	n.a.	core miopathy	TTN	chr2:179522230	c.T38033C	p.V12678A	het	
					TTN	chr2:179527095	c.C37009T	p.P12337S	het	
27/39s	M	CM	Sp	cftdm	COL6A1	chr21:47406897	c.C628G	p.R210G	het	
27/41s	F	CM	n.a.	cftdm	SYNE1	chr6:152746682	c.G5001T	p.A1701S (spl.)	het	
					SYNE2	chr14:64484328	c.G4903A	p.E1635K	het	
27/42s	F	CM	n.a.	multiminicores	COL6A1	chr21:47406559	c.G548A	p.G183D	het	
					MYH7	chr14:23885359	c.G4807C	p.A1603P	het	
					DNM2	chr19:10909210	c.A1384G	p.T462A	het	
28/40s	M	CM	n.a.	n.a.	TTN	chr2:179415978	c.G91280T	p.G30427V	het	
					TTN	chr2:179415952	c.C91306T	p.R30436W	het	
28/41s	M	CM	Sp	d.f.	COL6A1	chr21:47410893	c.G1057A	p.G353S	het	
29/38s	M	LGMD	Rec	d.f.	COL6A2	chr21:47539756	c.G1324T	p.G442W	het	
					COL6A2	chr21:47551934*	c.G2528A	p.R843Q	het	AVSD^sr44^
30/38s	F	CM	Sp	n.a.	TTN	chr2:179411904	c.C94348T	p.R31450C	het	
					TTN	chr2:179428049	c.G82814A	p.G27604S	het	
31/39s	M	CM	Sp	minicores	ATP7A	chrX:77301920	c.G4356C	p.L1452F	het	
31/40s	F	CM	Sp	cftdm	PHKA1	chrX:71840734	c.G1978A	p.V660I	het	
31/41s	M	CM	Sp	reducing body	KBTBD13	chr15:65369638	c.C485T	p.T162M	het	
32/40s	M	CM	Sp	T1FP	TTN	chr2:179583104	c.C24729A	p.C8243X	het	
					TTN	chr2:179589034	c.A21068C	p.Q7023P	het	
33/38s	F	LGMD	Sp	d.f.	CNTN1	chr12:41337835	c.A1546G	p.I516V	het	
34/38s	F	LGMD	Sp	d.f.	SMCHD1	chr18:2656250	c.G176T	p.C59F	het	
34/41s	M	CM	n.a.	m.f.	COL6A2	chr21:47545473	c.C1911G	p.F637L	het	
35/41s	M	CM	n.a.	c.n.	DYSF	chr2:71730384	c.277G>A	p.A93T	hom	
					TTN	chr2:179411050	c.C95008T	p.R31670X	het	
36/38s	M	LGMD	Sp	d.f.	SYNE1	chr6:152651958	c.C15746T	p.T5249M	het	
36/39s	F	CM	Sp	cftdm	COL6A2	chr21:47545885	c.G2156A	p.R719Q	het	
					CPT1B	chr22:51012938	c.G767A	p.R256H	het	
36/40s	M	LGMD and DCM	Sp	m.f.	SYNE2	chr14:64447788	c.A1733G	p.K578R	het	
37/38s	M	LGMD	Sp	m.f.	COL6a3	chr2:238277282	c.A4824T	p.R1608S	het	

* Already reported. For references, see Additional file 10.

The most surprising finding was, however, the presence of additional damaging or potential damaging variants in 16 patients of the first two groups (23/108=21%) in whom other pathogenic variants or variants of uncertain significance had already been identified. These variants, if they had been detected alone in the context of a single gene testing, would have been considered as causative.

The third group includes 26 patients (26/177=15%) in which we discovered a single truncating variant (or a known disease-associated variant) in a recessive gene that is compatible with the phenotype. The second allele may carry a RNA splicing defect that is generally not predictable by DNA sequencing or, also, a variation in not investigated promoters or regulatory regions.

## Discussion

In the last decade, a remarkable progress has been made in discovering new disease genes and differentiating similar muscle disorders [1],[2]. This growing genetic heterogeneity highlights the problem of a very complex diagnosis [35]. Furthermore, genome sequencing studies suggest that the clinical genetic test may be incomplete not only when the causative mutation is missing, but also when the genotype/phenotype correlation appears weak. This is particularly true when the familial recurrence is unclear, with some relatives that only share minor affections. In families with patients who are more severely affected, this “grey area” is problematic for both genetic counselling and forthcoming mutation-specific treatments. However, this represents the proper challenge for the new genomic, high-throughput technologies: the power of discovery has been dramatically boosted by the introduction of the next-generation sequencing (NGS) techniques [13],[36]-[38]. In the NGS era, the genetic testing is going to move from few candidate genes to broader panels of genes [39] or, ultimately, to the entire genome. This will have consequences on the diagnostic flowchart: NGS tests may represent the first tier test, preceding biopsy and other invasive procedures.

We have applied both WES and targeted approaches to the diagnosis of genetic disorders of muscle and collected DNA samples of patients without diagnosis and realized that NGS technology can be helpful for clinical diagnostics, provided that a suitable tool is created. We traced an ideal profile of it. This tool should fulfil the following requirements [16],[20]: 1) to be cost-effective and thus applicable to a large number of patients and normal individuals, 2) to be robust in the terms of target reproducibility, 3) to be specific and sensitive with a limited need for further validation steps, 4) to be large enough to include all relevant genes and, finally, 5) to be easily upgradable in view of novel discoveries. Here we demonstrate the ability to generate this complex targeting and to fulfil all these requirements. We decided to use Haloplex as the enrichment technology. Haloplex first digests DNA using eight different combinations of endonucleases. Our experience suggests that this approach is more reproducible and accurate than the random mechanical DNA fragmentation. In addition, the capture is independent of the target base composition and is predictable from the probe design phase. As a proof of specificity and efficiency, we show that less than 2% of reads generated by Motorplex are off-target, in comparison with >12% of WES. This factor further improves the cost-effectiveness of the approach. This platform, based on eight different digestions and hybridization, is more accurate, reproducible and sensitive in comparison with other published methods [34]. We have designed the MotorPlex to detect variations in 93 muscle-disease genes and assayed 177 pre-screened DNA samples from myopathic patients. It is important to consider that these are all patients with zero mutations so far detected, even if most of them have been lengthily studied using a gene-by-gene sequencing approach. The high coverage and depth obtained permitted us to detect variations in most genes with sensitivity comparable with Sanger sequencing. According to our conservative NGS data interpretation, in 52 patients (29%) the diagnosis is complete. However, the detection rate will grow after a further molecular characterization of putative pathogenic variations in a second group of 56 patients. In addition, there are 26 samples (15%) that have defects in one single allele associated with a recessive condition. We predict that most of these can carry an elusive hit on the other allele such as splicing defects or copy number mutation(s). A percentage of 15%, in fact, is a usual value for disease-causing variants not detectable by sequencing.

The most interesting and quite surprising finding is, however, the very high number of rare damaging variants identified and first the cases (26/177) with more damaging variants in other genes in addition to those classified as causative. These additional variants may have a potential modifier effect. This percentage of these genetically complex patients may be higher, if we consider that many other important muscular genes (even if not disease-causing) can also carry damaging alleles. We can easily predict that a broader NGS approach could strengthen this observation. We hypothesize that the intrafamilial and interfamilial phenotypic differences may be frequently related to the combinations of multiple disease-causing alleles, more than to SNPs or CNVs. The so-called “modifier gene variants” could be individually rare, but collectively common. A comprehensive view of all the genes involved in a pathological process helps to point out these alleles having a minor but probably not negligible role in the disease aetiology.

The ultimate goal of MotorPlex is given by the pooling performances. The specificity and sensitivity values are very high and quite similar to those obtained in singleton testing and, above all, the diagnostic rate is not affected. The potential applications of pooling are just in large studies of complex and non-Mendelian disorders when a large number of samples have to be analyzed to improve the statistical power [40]. Considering our finding of multiple damaging variants in disease genes, these large studies are just around the corner. In addition, MotorPlex may discover low-allelic fraction variants in single samples, as in somatic mosaicisms. The pooled MotorPlex is likewise the cheapest genetic test (Table 3) ever presented that is able to screen 93 complex conditions at the cost of a few PCR reactions.

**Table 3:** **Predicted enrichment costs and workload for single and pooled DNA samples**

Technical step	Cost (€)	
	Single	PoolSeq
Haloplex Kit (96 samples)	16240,83	4263,22
Polymerase	86	22,575
AMPure XP beads	400	105
Validation and quantification of enriched target DNA	386,8	101,5
Total (total per sample)	17113.63 (213.92)	4492.29 (56.15)
**Run Time**	**Total Time** (**h**)	
	Single	PoolSeq
Enrichment procedure	4days	1day

In conclusion, we here demonstrate that MotorPlex can be used to identify accurately all DNA variants also in huge muscle genes: the platform overcomes for sensitivity and coverage the WES approach. In addition, Pool-Seq may be the first option to perform cost-effective population studies to understand polygenic conditions. We think that similar protocols could be designed to extend the NGS applications to other studies for human genetics, as well as for disease prevention, nutrition, forensics and many others.

## Additional files

## Electronic supplementary material

Additional file 1: Table S1.: List of genes. (XLS 32 KB)

Additional file 2: Table S2.: Training set samples. (XLS 30 KB)

Additional file 3: Table S4.: Run Statistics of PoolSeq experiments. (XLS 23 KB)

Additional file 4: Figure S1.: Coverage comparison. (PPT 334 KB)

Additional file 5: Table S3.: Control variants. (XLS 32 KB)

Additional file 6: Table S5.: Summary of variants identified in training set samples. (XLS 22 KB)

Additional file 7: Figure S2.: Pooling strategy. (PPT 631 KB)

Additional file 8: Table S6.: Summary of rare variants identified in PoolSeq experiments. (XLS 40 KB)

Additional file 9: Figure S3.: Scout pools help filtering results. (PPT 144 KB)

Additional file 10: List of references for Table 1 and Table 2. (DOC 27 KB)

Below are the links to the authors’ original submitted files for images.

Authors’ original file for figure 1

Authors’ original file for figure 2

## Abbreviations

NGS: Next-generation sequencing
WES: Whole-exome sequencing
WGS: Whole-genome sequencing
IGV: Integrative genomics viewer
CM: Congenital myopathies
LGMD: Limb-girdle muscular dystrophy
FSHD: Facioscapulohumeral muscular dystrophy
EDMD: Emery-Dreifuss muscular dystrophy
DCM: Dilated cardiomyopathy
MH: Malignant hyperthermia
ARVD: Arrhythmogenic right ventricular cardiomyopathy
CMD: Congenital Muscular Dystrophy
HCM: Hypertrophic cardiomyopathy
VLCAD: Very long chain acyl-CoA dehydrogenase deficiency
CCD: Central core disease
AVSD: Atrioventricular septal defect
n.a.: not available
Sp: Sporadic
Rec: Recessive
Dom: Dominant
c.n.: Central nuclei
m.f.: Myopathic features
d.f.: Dystrophic features
cftdm: Congenital Myopathy with Fiber-Type Disproportion
T1FP: Type 1 fiber predominance
