# Supplementary material for: MotorPlex provides accurate variant detection across large muscle genes both in single myopathic patients and in pools of DNA samples
Source: Acta Neuropathol Commun. 2014 Sep 11;2:100. doi: 10.1186/s40478-014-0100-3 (PMC4172906; doi:10.1186/s40478-014-0100-3)
Supplement: Supplementary file 4 — Additional file 4: Figure S1.: Coverage comparison. (PPT 334 KB) [file 40478_2014_9100_MOESM4_ESM.ppt]

## Slide 1
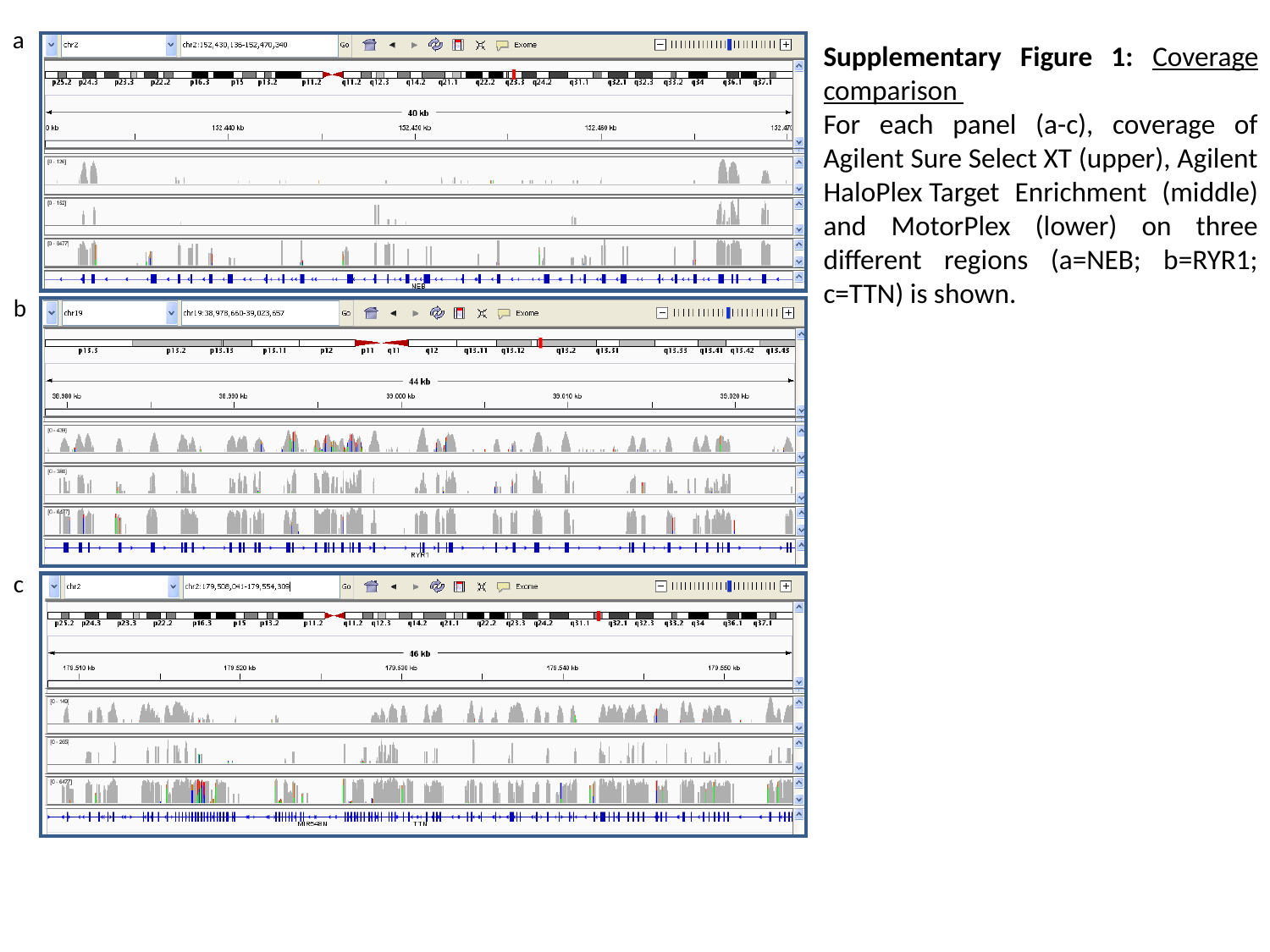

a
Supplementary Figure 1: Coverage comparison
For each panel (a-c), coverage of Agilent Sure Select XT (upper), Agilent HaloPlex Target Enrichment (middle) and MotorPlex (lower) on three different regions (a=NEB; b=RYR1; c=TTN) is shown.
b
c
