# Supplementary material for: MotorPlex provides accurate variant detection across large muscle genes both in single myopathic patients and in pools of DNA samples
Source: Acta Neuropathol Commun. 2014 Sep 11;2:100. doi: 10.1186/s40478-014-0100-3 (PMC4172906; doi:10.1186/s40478-014-0100-3)
Supplement: Supplementary file 7 — Additional file 7: Figure S2.: Pooling strategy. (PPT 631 KB) [file 40478_2014_9100_MOESM7_ESM.ppt]

## Slide 1
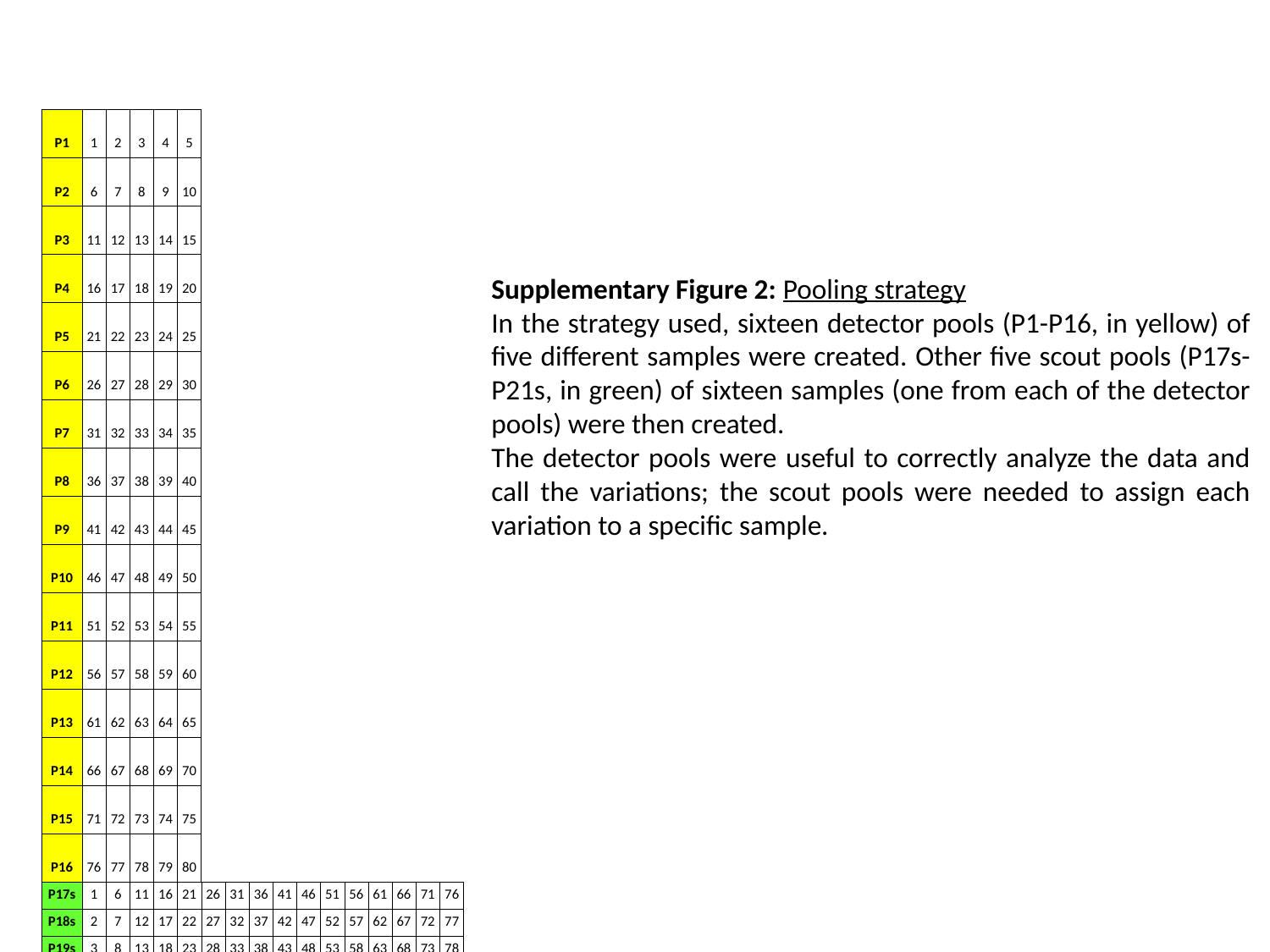

| P1 | 1 | 2 | 3 | 4 | 5 | | | | | | | | | | | |
| --- | --- | --- | --- | --- | --- | --- | --- | --- | --- | --- | --- | --- | --- | --- | --- | --- |
| P2 | 6 | 7 | 8 | 9 | 10 | | | | | | | | | | | |
| P3 | 11 | 12 | 13 | 14 | 15 | | | | | | | | | | | |
| P4 | 16 | 17 | 18 | 19 | 20 | | | | | | | | | | | |
| P5 | 21 | 22 | 23 | 24 | 25 | | | | | | | | | | | |
| P6 | 26 | 27 | 28 | 29 | 30 | | | | | | | | | | | |
| P7 | 31 | 32 | 33 | 34 | 35 | | | | | | | | | | | |
| P8 | 36 | 37 | 38 | 39 | 40 | | | | | | | | | | | |
| P9 | 41 | 42 | 43 | 44 | 45 | | | | | | | | | | | |
| P10 | 46 | 47 | 48 | 49 | 50 | | | | | | | | | | | |
| P11 | 51 | 52 | 53 | 54 | 55 | | | | | | | | | | | |
| P12 | 56 | 57 | 58 | 59 | 60 | | | | | | | | | | | |
| P13 | 61 | 62 | 63 | 64 | 65 | | | | | | | | | | | |
| P14 | 66 | 67 | 68 | 69 | 70 | | | | | | | | | | | |
| P15 | 71 | 72 | 73 | 74 | 75 | | | | | | | | | | | |
| P16 | 76 | 77 | 78 | 79 | 80 | | | | | | | | | | | |
| P17s | 1 | 6 | 11 | 16 | 21 | 26 | 31 | 36 | 41 | 46 | 51 | 56 | 61 | 66 | 71 | 76 |
| P18s | 2 | 7 | 12 | 17 | 22 | 27 | 32 | 37 | 42 | 47 | 52 | 57 | 62 | 67 | 72 | 77 |
| P19s | 3 | 8 | 13 | 18 | 23 | 28 | 33 | 38 | 43 | 48 | 53 | 58 | 63 | 68 | 73 | 78 |
| P20s | 4 | 9 | 14 | 19 | 24 | 29 | 34 | 39 | 44 | 49 | 54 | 59 | 64 | 69 | 74 | 79 |
| P21s | 5 | 10 | 15 | 20 | 25 | 30 | 35 | 40 | 45 | 50 | 55 | 60 | 65 | 70 | 75 | 80 |
Supplementary Figure 2: Pooling strategy
In the strategy used, sixteen detector pools (P1-P16, in yellow) of five different samples were created. Other five scout pools (P17s-P21s, in green) of sixteen samples (one from each of the detector pools) were then created.
The detector pools were useful to correctly analyze the data and call the variations; the scout pools were needed to assign each variation to a specific sample.
