# Supplementary material for: MotorPlex provides accurate variant detection across large muscle genes both in single myopathic patients and in pools of DNA samples
Source: Acta Neuropathol Commun. 2014 Sep 11;2:100. doi: 10.1186/s40478-014-0100-3 (PMC4172906; doi:10.1186/s40478-014-0100-3)
Supplement: Supplementary file 9 — Additional file 9: Figure S3.: Scout pools help filtering results. (PPT 144 KB) [file 40478_2014_9100_MOESM9_ESM.ppt]

## Slide 1
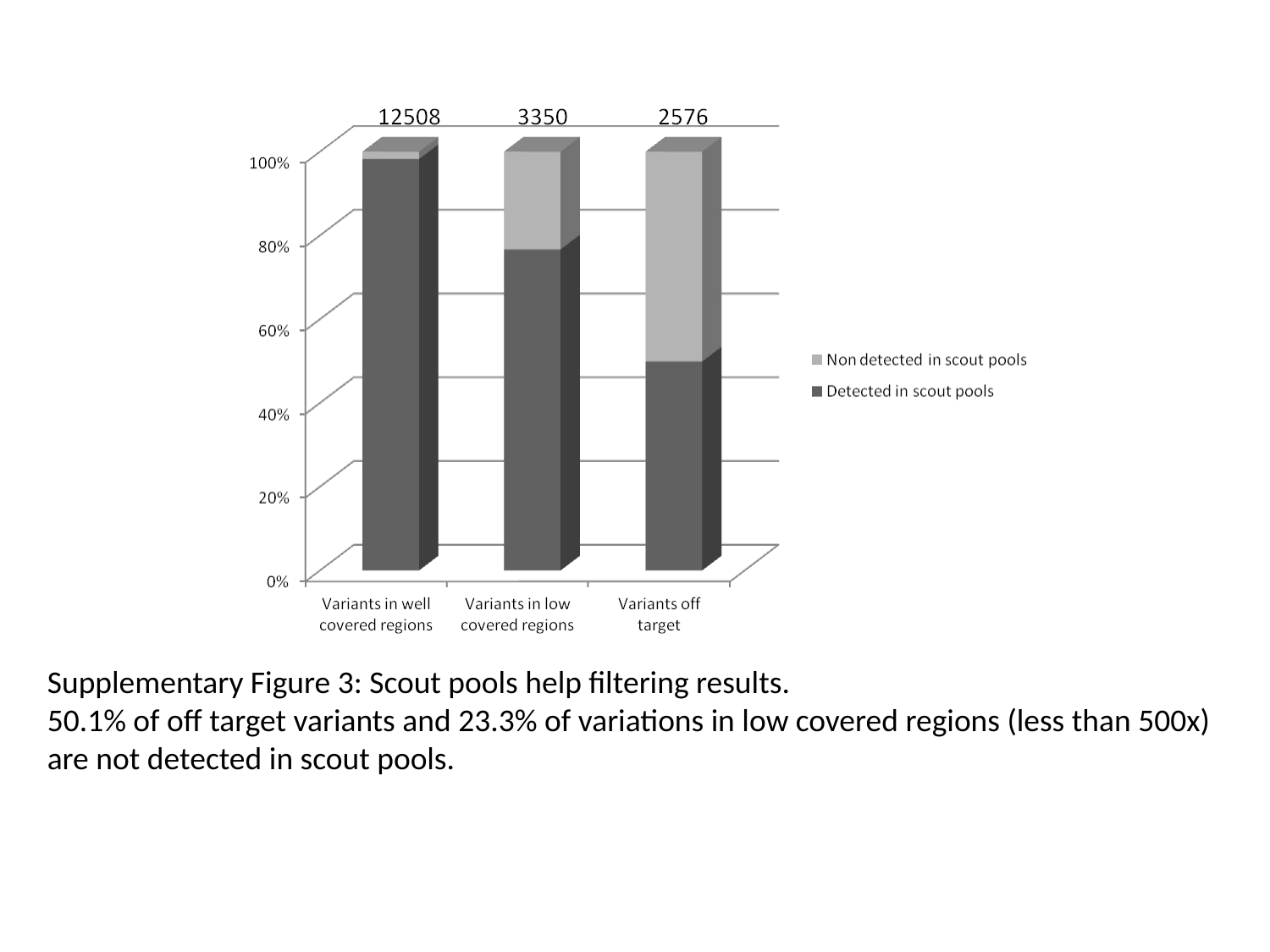

Supplementary Figure 3: Scout pools help filtering results.
50.1% of off target variants and 23.3% of variations in low covered regions (less than 500x) are not detected in scout pools.
