# Supplementary material for: MotorPlex provides accurate variant detection across large muscle genes both in single myopathic patients and in pools of DNA samples
Source: Acta Neuropathol Commun. 2014 Sep 11;2:100. doi: 10.1186/s40478-014-0100-3 (PMC4172906; doi:10.1186/s40478-014-0100-3)
Supplement: Supplementary file 10 — Additional file 10: List of references for Table 1 and Table 2. (DOC 27 KB) [file 40478_2014_9100_MOESM10_ESM.doc]

1 Bitoun M et al., Ann Neurol. 2007 Dec;62(6):666-70

2 Canki-Klain N et al., Am J Med Genet A. 2004 Mar 1;125A(2):152-6

3 Piluso G et al., J Med Genet. 2005 Sep;42(9):686-93

4 McNally EM et al., Hum Mol Genet. 1996 Nov;5(11):1841-7

5 Vatta M et al., J Am Coll Cardiol. 2003 Dec 3;42(11):2014-27

6 Klein A et al., Arch Neurol. 2011 Sep;68(9):1171-9

7 Duarte ST et al., Muscle Nerve. 2011 Jul;44(1):102-8

8 Tammaro A et al., Clin Genet. 2011 May;79(5):438-47

9 Gillies RL et al., Anaesth Intensive Care. 2008 May;36(3):391-403

10 Kostareva A et al., Acta Myol. 2011 Jun;30(1):42-5

11 Taylor M et al., Circulation. 2011 Aug 23;124(8):876-85

12 Piccolo F et al., Nat Genet. 1995 Jun;10(2):243-5

13 Carrié A et al., J Med Genet. 1997 Jun;34(6):470-5

14 Messina S et al., Neuromuscul Disord. 2008 Jul;18(7):565-71

15 Godfrey C et al., Brain. 2007 Oct;130(Pt 10):2725-35

16 Davidson AE et al., Brain. 2013;136(2):508-521

17 Bolduc V et al., Am J Hum Genet. 2010 Feb 12;86(2):213-21

18 Hayashi T et al., Biochem Biophys Res Commun. 2004 Jan 2;313(1):178-84

19 Spiekerkoetter U et al., J Pediatr. 2003 Sep;143(3):335-42

20 De Paula F. et al., Eur J Hum Genet. 2002 Dec;10(12):825-32

21 Richard I et al., Cell. 1995 Apr 7;81(1):27-40

22 Scharner J et al., Hum Mutat. 2011 Feb;32(2):152-67

23 Sacconi S et al., Am J Hum Genet. 2013 Oct 3;93(4):744-51

24 Vieitez I et al., Neuromuscul Disord. 2011 Dec;21(12):817-23

25 Naom I et al., Neuromuscul Disord. 1998 Oct;8(7):495-501

26 Piccolo F et al., Hum Mol Genet. 1996 Dec;5(12):2019-22

27 Spitali P. et al., Hum Mutat. 2009 Nov;30(11):1527-34

28 Meredith C et al., Am J Hum Genet. 2004 Oct;75(4):703-8

29 Sáenz A et al., Brain. 2005 Apr;128(Pt 4):732-42

30 Submitted to Leiden Database by M. Marttila

31 Zullo A et al, Hum Mutat. 2009 Apr;30(4):E575-90

32 Barone V et al., J Med Genet. 1999 Feb;36(2):115-8

33 Laing NG et al., Hum Mutat. 2009 Sep;30(9):1267-77

34 Davis MR et al., Neuromuscul Disord. 2003 Feb;13(2):151-7

35 Laporte J et al., Hum Mutat. 2000;15(5):393-409

36 Gillard EF et al., Genomics. 1991 Nov;11(3):751-5

37 Lynch PJ et al., Proc Natl Acad Sci U S A. 1999 Mar 30;96(7):4164-9

38 Magri F et al., BMC Med Genet. 2011 Mar 11;12:37

39 Zhang Q et al., Hum Mol Genet. 2007 Dec 1;16(23):2816-33

40 Manning BM et al., Am J Hum Genet. 1998 Mar;62(3):599-609

41 Tezak Z et al., Hum Mutat. 2003 Feb;21(2):103-11

42 Robinson RL et al., Hum Mol Genet. 1997 Jun;6(6):953-61

43 Fokstuen S et al., Hum Mutat. 2008 Jun;29(6):879-85

44 Ackerman C et al., Am J Hum Genet. 2012 Oct 5;91(4):646-59

45 Lampe AK et al., J Med Genet. 2005 Feb;42(2):108-20

46 Brito D et Madeira H, Rev Port Cardiol. 2005 Sep;24(9):1137-46

47 Cardim N et al., Rev Port Cardiol. 2005 Dec;24(12):1463-76

48 Krahn M et al., Hum Mutat. 2009 Feb;30(2):E345-75
