# Supplementary figures and images for: MotorPlex provides accurate variant detection across large muscle genes both in single myopathic patients and in pools of DNA samples
Source: Acta Neuropathol Commun. 2014 Sep 11;2:100. doi: 10.1186/s40478-014-0100-3 (PMC4172906; doi:10.1186/s40478-014-0100-3)

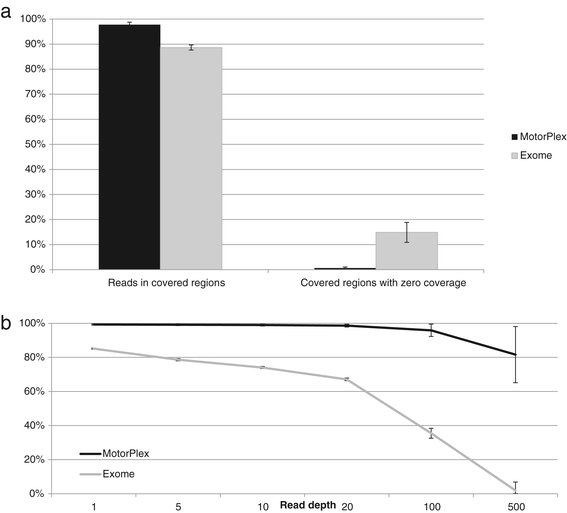

Supplement: Supplementary file 11 — Authors’ original file for figure 1 [file 40478_2014_9100_MOESM11_ESM.gif]

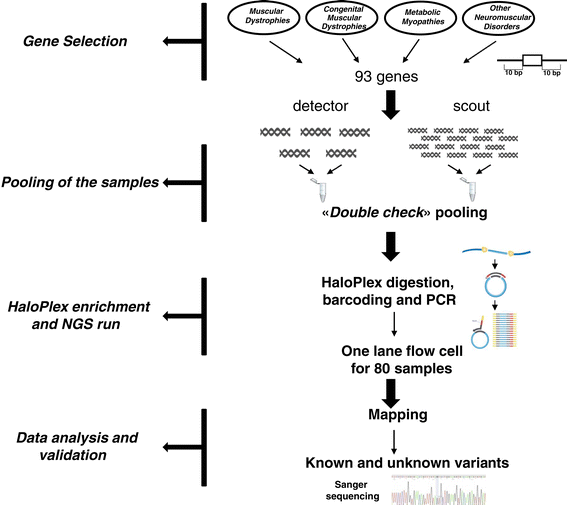

Supplement: Supplementary file 12 — Authors’ original file for figure 2 [file 40478_2014_9100_MOESM12_ESM.gif]
